# Early Diagnosis of Ectopic Pregnancy

**Published:** 1912-02

**Authors:** 


Early Diagnosis of Ectopic Pregnancy.—R. R. Huggins of Pittsburg (*Am. Jour. Obstet.* November, 1911), believes that the diagnosis of ectopic pregnancy should be made previous to the final rupture and collapse in at least 80 per cent. of the cases. The patient's history for a number of years previous to the development of symptoms should receive careful study. In a great majority of cases there has been a tubal infection of which a period of sterility is strong evidence. The menstrual history is very important. When a woman who has been regular goes beyond her time she will suspect pregnancy. If a flow from the vagina begins in a period varying from four or five days to three weeks after the regular time, continuing more or less regularly, accompanied by pains periodic in character located in the hypogastrium or on either side, extrauterine pregnancy should be suspected at once. In 95 per cent. of the cases of extopic pregnancy seen by the writer, the history and local symptoms have overshadowed all others, and seldom in the early weeks have the usual general signs been present. He shows how to differentiate between this condition and abortion, or salpingitis, or other condition which might be mistaken for it. He submits the histories of fourteen cases where the diagnosis was made and operation performed previous to the time of rupture.

